# Fabrication of Flexible Piezoelectric PZT/Fabric Composite

**DOI:** 10.1155/2013/914380

**Published:** 2013-11-18

**Authors:** Caifeng Chen, Daiwei Hong, Andong Wang, Chaoying Ni

**Affiliations:** ^1^School of Materials Science and Engineering, Jiangsu University, Zhenjiang, Jiangsu 212013, China; ^2^Department of Materials Science and Engineering, University of Delaware, Newark, DE 19716, USA

## Abstract

Flexible piezoelectric PZT/fabric composite material is pliable and tough in nature which is in a lack of traditional PZT patches. It has great application prospect in improving the sensitivity of sensor/actuator made by piezoelectric materials especially when they are used for curved surfaces or complicated conditions. In this paper, glass fiber cloth was adopted as carrier to grow PZT piezoelectric crystal particles by hydrothermal method, and the optimum conditions were studied. The results showed that the soft glass fiber cloth was an ideal kind of carrier. A large number of cubic-shaped PZT nanocrystallines grew firmly in the carrier with a dense and uniform distribution. The best hydrothermal condition was found to be pH 13, reaction time 24 h, and reaction temperature 200°C.

## 1. Introduction

Piezoelectric materials have the ability to convert mechanical energy into electrical energy and have potential applications as smart materials for sensors or actuator devices which require high direction sensitivity in structural health monitoring. Lead zirconate titanate (PZT) is a common piezoelectric material which is commercially used for piezoelectric actuators and sensors. But the monolithic PZT piezoelectric ceramic materials, including PZT patches or wafers, are always very brittle, and their fatigue resistance is also poor, which makes them vulnerable to accidental breakage during handling and bonding procedures, as well as their extremely limited ability to be conformed to curved surfaces. Consequently, it also seriously affects the sensitivity of the sensor or actuator devices [1–3].

In resolving the inadequacies of monolithic piezoceramic materials, many achievements have been obtained in developing composite piezoelectric materials with high elasticity and piezoelectric properties [4–6]. Recently, Chen and coworkers fabricated a flexible 1–3 piezo-composite made up by PZT microfibers, the micro-PZT fibers were arranged along one direction within the epoxy resin matrix [7]. Qiu et al. introduced Pb(Nb,Ni)O_3_-Pb(Zr,Ti)O_3_(PNN-PZT) piezoelectric ceramic fibers with a metal core to strengthen the toughness of the ceramic fibers [8].Piezoelectric fiber composites with elastic coating were employed to prevent the piezoelectric materials from mechanical failure [9, 10].However, it is still difficult to improve the low fracture toughness and electric fatigue of this delicate type of material. Currently, a big challenge is to obtain PZT composite material with high soft and toughness.

In this study, details of fabrication of piezoelectric PZT/fabric composite material with high pliable properties using hydrothermal method were reported. The growth of the PZT piezoelectric crystalline on fabric carrier and the optimum hydrothermal conditions were also studied.

## 2. Experimental Process

According to the measurement of molecular formula Pb(Zr_0.52_Ti_0.48_)O_3_, this experiment used tetrabutyl titanate (Ti(OC_4_H_9_)_4_), lead nitrate (Pb(NO_3_)_2_), and zirconium oxychloride (ZrOCl_2_·8H_2_O) as raw materials. Tetrabutyl titanate was poured into the mixed solution of lead nitrate and zirconium oxychloride slowly. After magnetic mixing for 10 min, sodium hydroxide (NaOH) solution was dripped into the mixed liquor until achieved a certain pH value; then the mixed liquor was transferred into the hydrothermal synthesis reactor, with the filling factor of 60%. 3~5 tablets of glass fiber cloth carrier with suitable size were placed in the hydrothermal synthesis reactor; then the reactor was put in the oven at the temperature of 200°C for a certain reaction time. After natural cooling to room temperature, the carriers were washed with deionized water carefully, and 0.1 mol·L^−1^ silver nitrate (AgNO_3_) solution was used to test the liquid detergent until no chlorideion content was detected. Then dried the carriers at the temperature of 80°C for 2 h, the PZT/fabric piezoelectric composite material was obtained. Specific reaction conditions are shown in Table 1.

Microstructures of the composites and nano-PZT crystal particles were characterized with Scanning Electron Microscopy (SEM, JSM-7001F) and Energy Dispersive X-ray Detector (EDAX) techniques.

## 3. Results and Analysis

### 3.1. The Morphology Characteristics of PZT/Fabric Piezoelectric Composite

**Figure 1:**
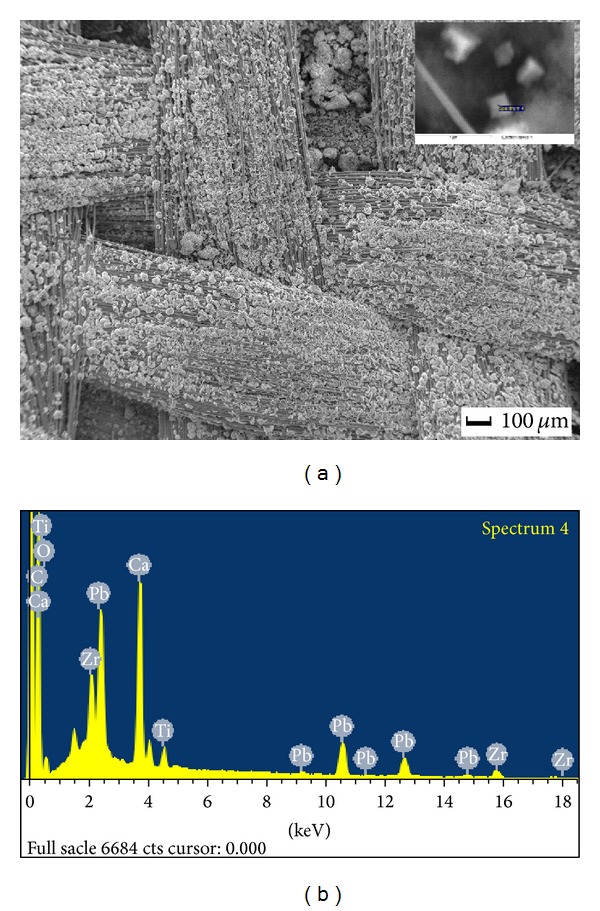
SEM morphology and energy dispersive X-Ray spectrum of the PZT/glass fabric piezoelectric composite (a) morphology of composite; (b) energy dispersive X-Ray spectrum.

A variety of fabrics as carriers were tested in the paper, such as cotton cloth, cloth filter, ceramic fiber cloth, and nonwoven fabrics. The results showed that most of the fabric materials were melted completely in the hydrothermal synthesis reactor when under high temperature, high pressure, and strong alkali corrosion conditions. Because ceramic fiber paper is adhered by glue, it cannot retain either. Although cotton cloth, nonwoven fabrics, and glass fiber cloth can remain in the environment of reaction kettle, cotton cloth is badly damaged and becomes ragged, and nonwoven will contract with great charge in dimension. Only the glass fiber cloth can keep the original fiber structure, and the integrity of fiber can be maintained without any breakage and its elasticity still well as shown in Figure 1(a).

From Figure 1(a), we can observe that many PZT nanocrystallines grow on the soft glass fiber cloth surface intensively. Grain is cubic shaped, as shown in the zoomed image of the upper right corner of Figure 1(a). As the spectrum analysis from Figure 1(b), the chemical composition of the grain contains Pb, Zr, Ti, and O elements; its composition and proportions are consistent with PZT material. Other elements like Ca and C, which can be seen in the spectrum, come from the composition of glass fiber cloth carrier.

### 3.2. pH Value on the Influence of the Nano-PZT Crystal Particles Growth

**Figure 2:**
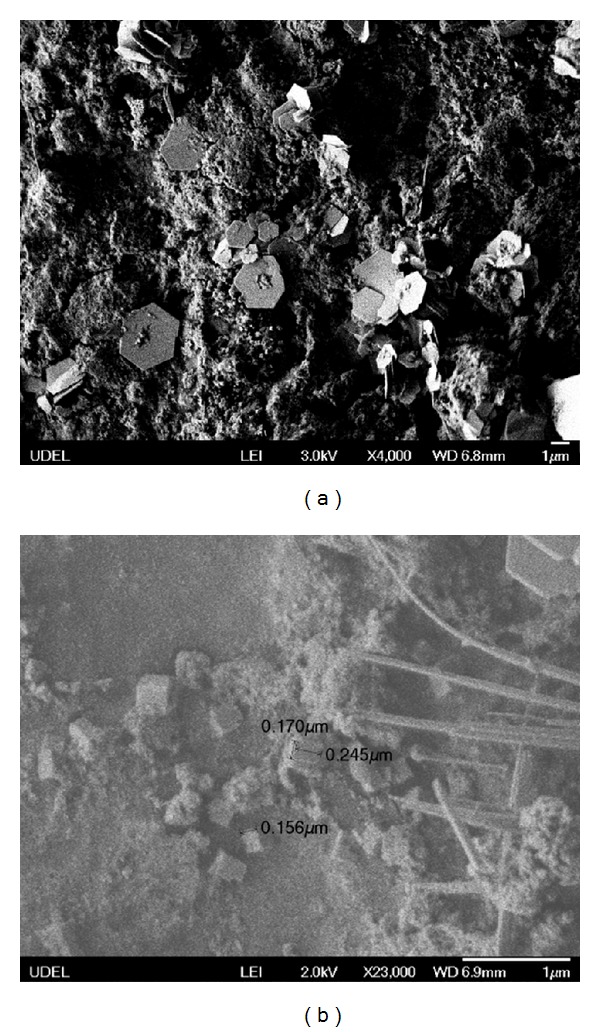
pH value on the influence of the PZT nanocrystalline morphology (a) pH = 8; (b) pH = 13.

In different pH value, the morphology of PZT nanocrystallines is different on the glass fiber cloth carrier. Figures 2(a) and 2(b) are the micromorphology of A1 and A2 samples when pH value is 8 and 13, respectively. In Figure 2(a) there appears the hexagon lamellar structure of the PZT nanocrystallines, while some fibrous and cubic-shaped PZT nanocrystallines can be seen in the Figure 2(b). 

It's well known that the crystal formation of PZT is perovskite structure, and its basic unit is oxygen octahedral structure. The oxygen octahedral structure can join into PZT crystal in the way of concurrent. If the main growth direction is in one-dimension, fibrous PZT crystal will generate, while the sheet-like or layered PZT crystal will generate if the major growth direction is in two-dimensional. The angular cubic-shaped PZT grain will generate if the main growth direction is in tridimensionality. 

In fact, alkaline condition is conducive to PZT grain crystallization and growth; the pH value affects the combination way of PZT crystal oxygen octahedral seriously. Pb^2+^ ions and OH^−^ ions combine in the growth process, and the growth direction of oxygen octahedral is mainly two-dimensional, while the growth direction of oxygen octahedral is exceptionally slow in three-dimensional direction when the pH value is low, thus, the PZT crystallization becomes sheet-like or layered, as shown in Figure 2(a). But when the PH value is higher, because the Na^+^ ion's radius is small, a lot of Na^+^ ions get into the precursor body of the hydrated ion of Zr and Ti, and PZT crystal precursor receive the impact of Na^+^ ions. It results in PZT crystal precursor's fracture and makes oxygen octahedral grow in tridimensional space with different degrees. The growth direction of the crystals is in tridimensional, and the crystals appear fibrous or cubic shapes, as shown in Figure 2(b).

### 3.3. Reaction Time on the Influence of the Nano-PZT Crystal Particles Growth

Pb(OH)_2_, Zr(OH)_4_, and Ti(OH)_4_ are the hydrate of lead, zirconium and titanium elements of the raw materials; they will be suspended in the mixture solution because of their different solubility. In alkaline conditions, when all these suspended reactant particles in the solution are partly dissolved in solution, they form supersaturated solution phase. It generates the PZT crystal nucleus with the interaction of supersaturated ion or ionic group, nucleus collides each other in the suspension, small crystal nucleus gradually dissolve, and big crystal nucleus grow up slowly then precipitate out from the solution to a certain degree.

In the hydrothermal system, adsorption and sedimentation of the growing basic unit on the crystal surface strongly affect the crystal growth rate and direction. Due to theslow speed of dissolution and precipitation, the reaction time affects the formation of the PZT crystal morphology greatly. When the reaction time is longer, the degree of precipitation will be deeper, and PZT crystal morphology characteristics are more obvious. 

**Figure 3:**
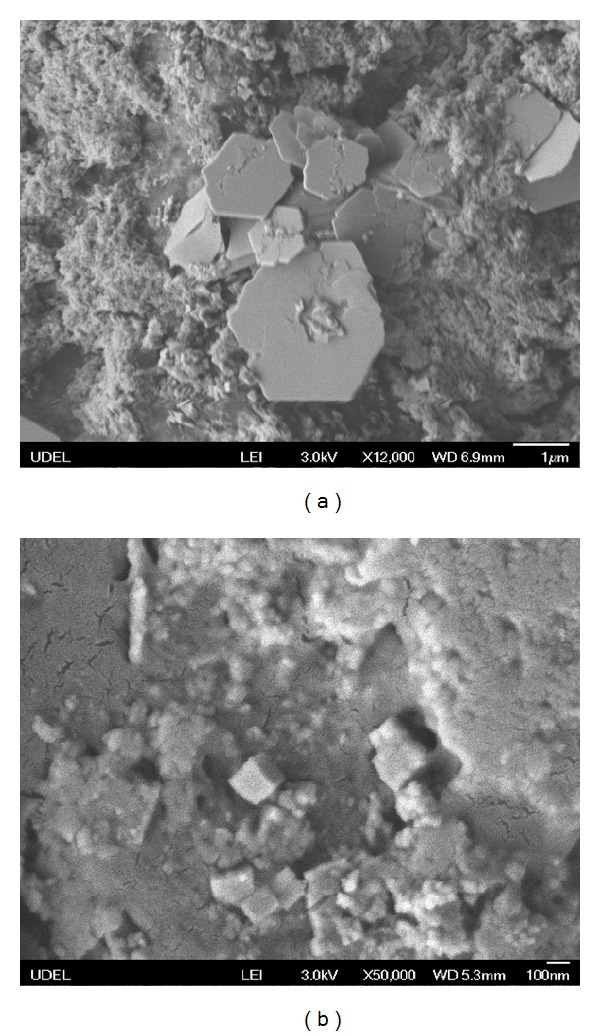
SEM morphology of PZT crystal in different reaction time (a) 10 h; (b) 18 h.

Sheet-shaped PZT nanocrystallines could generate in fiber glass carrier after the mixture reacted for 10 h, as shown in Figure 3(a), namely, sample A3. While after reaction for 18 h, the quality of sheet nanocrystalline on carrier began to reduce, a large number of cubic-shaped PZT nanocrystallines began to grow out, as shown in Figure 3(b), namely, sample A4. It can be concluded that a longer hydrothermal reaction time makes it easier to get cubic-shaped PZT nanoparticles with dissolving the precursors and absorbing reactive ion compound. 

## 4. Conclusion

Soft glass fiber cloth is an ideal kind of carrier, it can keep the original fiber structure and well elasticity, and the integrity of fiber can be maintained without any breakage in the environment of reaction kettle. It is relatively appropriate to prepare piezoelectric composite material, and a lot of PZT nanocrystallines can grow on the carrier firmly and intensively through hydrothermal method. During this process, pH value affects the morphology of the PZT crystal grain. When the pH value is less than 10, the PZT crystal is in lamellar structure, while cubic-shaped PZT crystal can generate when the pH value is 13. Therefore, good morphology of the PZT crystal can be obtained under pH = 13. The reaction time also affects the morphology of the PZT crystal grain seriously, as the reaction time increased, it is easier to get cubic-shaped PZT nanocrystallines. The reaction time influences both the integrity and the size of grain, and the best reaction time is 24 hours.

## Figures and Tables

**Table 1:** The hydrothermal conditions of each sample.

Sample	pH value	Hydrothermal time (h)
A1	8	24
A2	13	24
A3	13	10
A4	13	18

## References

[1] Okayasu M, Sato Y, Mizuno M, Shiraishi T (2012). Mechanically controlled domain structure in PZT piezoelectric ceramics. *Ceramics International*.

[2] Gallo CA, Tofoli FL, Rade D, Steffen V (2012). Piezoelectric actuators applied to neutralize mechanical vibrations. *Journal of Vibration and Control*.

[3] Smith GL, Pulskamp JS, Sanchez LM (2012). PZT-based piezoelectric MEMS technology. *Journal of the American Ceramic Society*.

[4] Chen C, Wang A, Han X, Ni C, Liu J (2012). Preparation and piezoelectric properties of PZT nano fibers and PZT textured ceramics. *Science of Advanced Materials*.

[5] Garcia-Gancedo L, Olhero SM, Alves FJ (2012). Application of gel-casting to the fabrication of 1–3 piezoelectric ceramic-polymer composites for high-frequency ultrasound devices. *Journal of Micromechanics and Microengineering*.

[6] Han X, Chen C, Wang A, Luo Y, Liu J, Ding Z (2012). Fabrication and characterization of micro piezoelectric fibers and 1–3 composites. *Nanoscience and Nanotechnology Letters*.

[7] Chen C, Han X, Liu J, Ding Z (2012). Fabrication and piezoelectric property characterization of new micro PZT fibers and 1–3 piezo-composites. *Nanoscience and Nanotechnology Letters*.

[8] Qiu JH, Tani J, Yamada N, Takahashi H Fabrication of piezoelectric fibers with metal core.

[9] Hashemi R, Weng GJ, Kargarnovin MH, Shodja HM (2010). Piezoelectric composites with periodic multi-coated inhomogeneities. *International Journal of Solids and Structures*.

[10] Maji S, Asrey R, Kumar S (2010). Polymer-coated piezoelectric quartz crystal sensor for sensing the nerve agent simulant dimethyl methylphosphonate vapor. *Journal of Applied Polymer Science*.

